# Parent/Carer-Reported Experience of Shared Decision Making at Child and Adolescent Mental Health Services: A Multilevel Modelling Approach

**DOI:** 10.3389/fpsyt.2021.676721

**Published:** 2021-07-15

**Authors:** Shaun Liverpool, Daniel Hayes, Julian Edbrooke-Childs

**Affiliations:** ^1^Evidence Based Practice Unit, University College London and Anna Freud National Centre for Children and Families, London, United Kingdom; ^2^Faculty of Health, Social Care and Medicine, Edge Hill University, Ormskirk, United Kingdom; ^3^Health Services and Population Research Department, King's College London Institute of Psychiatry, Psychology and Neuroscience, London, United Kingdom

**Keywords:** multilevel analysis, logistic regression, parents, child mental health, carer

## Abstract

**Background and Objective:** Shared decision making (SDM) has been associated with positive outcomes at child and adolescent mental health services (CAMHS). However, implementing SDM is sometimes challenging. Understanding the factors associated with parent/carer experience of SDM could provide empirical evidence to support targeted efforts to promote SDM. This study aimed to explore the frequency of parent/carer-reported experience of SDM and examine possible associations between SDM and clinician's perceptions of the (a) children's and young people's psychosocial difficulties, (b) additional complex problems, and (c) impact of the psychosocial difficulties.

**Methods:** Secondary analysis was conducted on administrative data collected from CAMHS between 2011 and 2015. The sample was composed of 3,175 cases across 58 sites in England. Frequencies were recorded and associations were explored between clinician-reported measures and parent/carer-reported experiences of SDM using a two-level mixed-effect logistic regression analytic approach.

**Results:** Almost 70% of parents/carers reported experiencing higher levels of SDM. Individual-level variables in model one revealed statistically significant (*p* <0.05) associations suggesting Asian parents/carers (OR = 1.95, 95% CI [1.4, 2.73]) and parents/carers having children with learning difficulties (OR = 1.45, 95% CI [1.06, 1.97]) were more likely to report higher levels of SDM. However, having two parents/carers involved in the child's care and treatment decisions (OR = 0.3, 95% CI [0.21, 0.44]) and being a parent/carer of a child or young person experiencing conduct problems (OR = 0.78, 95% CI [0.63, 0.98]) were associated with lower levels of SDM. When adjusting for service level data (model two) the presence of conduct problems was the only variable found to be significant and predicted lower levels of SDM (OR = 0.29, 95% CI [0.52, 0.58]).

**Conclusion:** Multilevel modelling of CAMHS administrative data may help identify potential influencing factors to SDM. The current findings may inform useful models to better predict and support SDM.

## Introduction

Shared decision making (SDM) is defined as the involvement of service users in the decision-making process where there are important competing care and treatment options (1, 2). This approach to health decisions has been widely advocated across various health settings and patient populations (3, 4). However, in child and adolescent mental health services (CAMHS), the SDM process is unique as it involves a sometimes-complex triad relationship between clinicians, parents/carers and children or young people (5, 6). Yet, previous studies have mainly focused on the dyad relationships between clinicians and adult patients (7). SDM in chronic care settings, like CAMHS, may require service users to make and revisit decisions, with fewer decisions occurring during the clinical encounter and several ongoing lifestyle decisions, compared to acute physical care (8). Therefore, the areas where triad relationships exist in chronic care settings have been less understood, with implication for a universally accepted definition (9). Consequently, it is vital to monitor SDM to ensure elements of SDM are still being met. Makoul and Clayman (10) described an SDM model with nine essential elements. These include identifying or discussing: the problem; treatment options; benefits/risks; service user values/preferences; service provider recommendations; service user understanding; service user abilities/self-efficacy; decisions; and arranging a follow-up. Nonetheless, some researchers indicate that passive involvement in SDM is quite common in pediatric care. A previous study evaluating videotapes of 101 child care visits to 1 of 15 physicians observed that around 65% of cases resulted in decision making efforts mainly from the physician and fewer cases with child or parent involvement (11).

### Frequency of Service-User-Reported SDM

Despite researcher observations, studies conducted in the USA suggest that many parents/carers (55–68%) generally report experiencing SDM in CAMHS, reporting mean scores of 3.37 to 3.6 out of a possible four on SDM outcome measures (12–15). These studies analyzed data from national surveys that explored physical health (e.g., asthma) and common mental health and behavioral conditions (e.g., attention deficit/ hyperactivity disorder, anxiety, depression, conduct problems and autism spectrum disorder) in children up to the age of 17 years. Based on the available datasets the authors used composite measures of SDM including questions such as “If there were a choice between treatments, how often would your medical provider ask you to help make the decision?” (14) or “How often did they [clinicians] make it easy for you to ask questions or raise concerns?” (12, 13). All previous authors acknowledged the absence of a validated parent/carer reported SDM measure as a key limitation. It was also noted that the inability to capture the views of the child or young person could have potentially influenced their findings (15).

Similar findings have been reported in youth physical health (16) and adult mental health settings (17). In Europe, a study including over 8,000 participants in the general population found that over half (51%) of the sample reported experiencing aspects of SDM (18). Around 71% of the English respondents reported being satisfied with their level of involvement and being involved as much as they wanted to (18). National surveys in England have also shown an upward trend (52–59%), with more patients reporting experiencing SDM in the last decade (19).

Nonetheless, a scoping review of parent-targeted SDM interventions in CAMHS reported that existing interventions met an average of 4.57 SDM elements (20). To achieve this, the authors conducted a mapping exercise using the Makoul and Clayman (10) SDM model of nine essential SDM elements to evaluate the identified decision support tools. That finding suggests there is still room to improve when providing support to parents/carers to promote SDM. There is also evidence suggesting that only about 50–55% of parents/carers report discussing child psychosocial difficulties with health professionals (21). Further, previous studies reported lower SDM among families with children experiencing mental health conditions compared to physical health conditions (15). Taken together, researchers may agree that our understanding of the extent to which parents/carers of children with psychosocial difficulties experience SDM when accessing care is still limited. Similarly, the existing evidence indicates that SDM may be influenced by several factors, including demographics and clinical characteristics.

### Potential Factors Influencing SDM

Studies in general healthcare report that younger patients and those with higher educational levels preferred involvement in SDM (22). Similarly, other population-based studies in the USA and Canada reported that younger persons and women experienced more involvement in SDM (23, 24). Researchers in physical health have also observed lower involvement in SDM opportunities from ethnic minority groups (25). In CAMHS, research suggests that higher levels of SDM are associated with children and young people (CYP) and parents/carers experiencing improvement in psychosocial difficulties (26). Similarly, higher SDM was associated with CYP experiencing mild mental health difficulties vs. those experiencing moderate to higher levels of difficulties or decreasing impairment scores (13, 14, 27). However, an in-depth understanding of parents/carers' involvement in SDM in CAMHS is still limited, as qualitative findings and observation reports suggest that parents/carers of children with psychosocial difficulties *struggle* to be involved in SDM (11, 28, 29). To support this group of parents, researchers are beginning to explore an affective appraisal approach for SDM in CAMHS. This model incorporates the emotional states of parents, by exploring a two-way direction that emotions may be influencing parents' involvement in SDM and vice versa (30).

### Rationale for the Current Study

The above evidence suggests that families of CYP with psychosocial difficulties may be at risk of experiencing varying levels of SDM. Studies thus far generally examined the association between SDM and parental perceptions of child mental health status highlighting limitations such as self-report bias. This can have implications for how findings are interpreted, as previous research shows a higher proportion of parents (41.6%) may recognize externalizing problems compared to internalizing symptoms (28.1 %) (31). In the same vein, parent/carer perceptions of psychosocial difficulties may differ from CYP's perceptions (32). Therefore, further studies representing an objective view of CYP's psychosocial difficulties (e.g., clinicians' perspective) can support the existing literature. In addition, previous studies focused mainly on specific psychosocial problems (e.g., severity or impairment) among children up to age 18 and failed to account for comorbidities (e.g., learning difficulties) and further complex problems, such as the parent's own health. Also, due to the complex nature of SDM, the growing interest in the field and the potential service user and service provider influencing factors, it is of great importance to identify target areas for improvement. Lastly, given that CYP generally appreciate the involvement of their parent/carer in treatment decisions (33–35), an examination of associations as potential barriers to parent/carer experience of SDM could also be beneficial.

### Aims

This study has three overarching aims. First, to explore the frequency of higher *quality* parent/carer-reported experience of SDM at CAMHS. Second, to examine associations between parent/carer-reported experience of SDM and clinicians' perceptions of the (a) presence of CYP's psychosocial difficulties, (b) presence of additional complex problems, and (c) impact of the psychosocial difficulties. Third, to investigate the potential influence of service level variables on parent/carer-reported experience of SDM.

## Methods

### Participants

A secondary analysis was conducted on administrative data routinely collected from clinicians and parents/carers accessing CAMHS; more specifically those accessing the Children and Young People's Improving Access to Psychological Therapies (CYP IAPT) between 2011 and 2015 (36). The sample included in the current study was composed of *N* = 3,175 cases of CYP accessing care from 58 CAMHS offered by the National Health Services in England. The CYP were between the ages of 0 and 23 years with a mean age of 11.08 (SD = 3.93) years at the point of data collection. The sample was predominantly White (68%), with approximately half the sample being parents/carers of girls (52%), and the majority of the sample being mothers (66%). Further details of the sample is included in Table 1.

**Table 1:** Characteristics of the sample.

**Characteristic**	***n* (%)**
**Demographics**	
Relationship to child	
Mother	2,084 (66)
Father	192 (6)
Both parents	790 (25)
Other	109 (3)
Age of child	
0 to 10	1,304 (41)
11 to <25	1,871 (59)
Ethnicity	
White	2,167 (68)
Mixed race	182 (6)
Asian	232 (7)
Black	150 (5)
Other	444 (14)
Gender of child	
Male	1,539 (48)
Female	1,636 (52)
**Psychosocial difficulties**	
Separation Anxiety	706 (22.24)
Social Anxiety	782 (24.63)
General Anxiety	845 (26.61)
^a^OCD	403 (12.69)
Panic disorder	511 (16.09)
Agoraphobia	358 (11.28)
Depression	796 (25.07)
Self-harm	448 (14.11)
^b^ADHD	440 (13.86)
Conduct disorders	507 (15.97)
Difficult to manage	588 (18.52)
Family problems	777 (24.47)
Attachment problems	496 (15.62)
Peer problems	757 (23.84)
Other	1,824 (57.45)
**Additional problems**	
Learning disabilities	283 (8.91)
Autism	375 (11.81)
Child in need	218 (6.87)
Experience of abuse	395 (12.44)
Parental health issues	704 (22.17)
Financial difficulties	238 (7.50)
Other	614 (19.34)
**Impact on CYP**	
Home	833 (26.24)
School/work	796 (25.07)
Community	488 (15.37)
Service engagement	261 (8.22)

a *Attention-deficit and hyperactivity disorders;*

b *Obsessive compulsivity disorders; M, Mean; SD, Standard deviation; CYP, Children and young people*.

*N = 3,175 (n refers to the count for each condition). Percentages representing psychosocial difficulties, additional problems and impact may not total 100 due multiple responses for each case*.

### Measures

#### Covariates

##### Demographic Characteristics

We included the CYP's gender, age and ethnicity as covariates. Gender was categorized as male, female or other. Age was measured on a continuous scale. Ethnicity was recorded using the 2001 Census classification (37), and based on self-report by the young person or their parent/carer. For the purpose of analysis, ethnicity was collapsed into five broad categories: White, Mixed, Asian, Black and Other ethnic groups. The relationship to the child or young person was categorized as father, mother, both parents, and other to reflect the person (s) completing the SDM measure. The anonymised site identifier was used to denote the different CAMHS site the families attended.

#### Criterion Variables

The Current View Tool (CVT) is a clinician-reported measure that routinely captures information about a child or young person and their family. The clinician utilizes information from meetings with the CYP and their families, pre-meeting liaison (e.g., referrals, teachers and other health professional notes), patient-reported outcomes measures and clinician-rated measures (38).

The CVT records 30 presenting problems, 14 additional complex problems, as well as six contextual problems (e.g., impact on the school or home) and issues in education, employment or training. Generally, the ratings of the CVT do not imply a diagnosis (38, 39). However, routinely collected data have several strengths including comprehensiveness, cost-effectiveness and the ability to capture the same data throughout the National Health Services (NHS) allowing for comparison (40). The items on the CVT were used to assess the presence of psychosocial difficulties and complex problems as well as the impact of these problems.

##### Presence of CYP Psychosocial Difficulties

To assess the presence of psychosocial difficulties, 30 items of the CVT were used. Items included responses to statements such as “Anxious away from home,” “Depression/low mood” and “Eating issues.” Responses to the psychosocial items on the CVT were rated on a five-point scale with the responses categorized as “None,” “Mild,” “Moderate,” “Severe,” and “Not known.” To capture the presence/absence of psychosocial difficulties, the responses “None” and “Not known” were coded as 0 and labeled as condition “absent or unknown.” The decision to group these together was based on the assumption that the clinician had insufficient information to even identify mild symptoms. It was also observed that the unknown category represented <10% of the total sample. All other responses were coded as one and labeled as condition present.

Items with low frequencies (i.e., those representing <10% of the sample) were grouped together in a single category and labeled “Other” to avoid including under-powered groups in the main analysis. This group included items such as Gender Identity Disorder, Selective mutism and Substance abuse which clinicians reported on fewer occasions. As a result, 14 distinct problem types were represented in addition to “Other” totalling to 15 categories.

##### Presence of Complex Problems

To assess the presence of complex problems, 14 items of the CVT were used, capturing the presence of different factors, such as “Looked after child,” “Parental issues” and “Deemed child in need of social services input.” Responses were categorized as “Yes,” “No” and “Not known.” To capture the presence/absence of additional complex problems; the responses “No” and “Not known” were coded as 0 and labeled as condition “absent or unknown,” and “Yes” to any of the items was coded as 1 and labeled as present. Similar to psychosocial difficulties, the additional complex problems with low frequency (e.g., having current protection plan and contact with the justice system) were grouped into a category called “Other” resulting in seven possible categories of additional complex problems.

##### Impact of Psychosocial Difficulties

To capture the impact of the psychosocial difficulties, items describing four contextual problems were used (i.e., difficulties at home; school, work or training; community and service engagement). Responses to the impact items were also rated on a five-point scale with the response categorized as “None,” “Mild,” “Moderate,” “Severe,” and “Not known.” To capture the impact, responses “None” and “Not known” were coded as zero and labeled as “absent or unknown” and all other responses were coded as one and labeled as present.

#### Outcome Variable

##### Parent/Carer-Reported Experience of SDM

To measure parent/carer-reported experience of SDM using the available measures collected in the dataset, the following four items of the Experience of Service Questionnaire [ESQ, (41)] were used: (1) I feel that the people who have seen my child listened to me; (2) It was easy to talk to the people who have seen my child; (4) My views and worries were taken seriously and (6) I have been given enough explanation about the help available here. Previous studies have also utilized these items as a composite score for SDM (26). Responses to these questions were dichotomized and coded as Yes = 1 and No = 0. For the purpose of this research, an overall composite score of the four items were tallied and a parent/carer with a total score of 4 was classed as experiencing higher levels (i.e., quality) of SDM and any value <4 was classed as experiencing lower levels of SDM. Previous researchers have also utilized similar approaches to discriminate between levels of SDM (15). The four-item SDM measure displayed high internal consistency (Cronbach's alpha 0.9) with the current sample.

#### Design and Statistical Analysis

##### Preliminary Tests

To ascertain whether Logistic Regression models could be used for our analysis and to ensure the validity of the data, all assumptions were tested. The sample size of 3,175 was deemed adequate given the number of predictor variables (42). The assumption of no multicollinearity was also met. All Variance Inflation Factor (VIF) scores were <5 with a mean VIF of 1.57 implying that none of the independent variables correlated highly with each other (43). All potential outliers were removed prior to analysis (44).

##### Main Analysis

First, descriptive data including frequencies of SDM was calculated. Then we investigated the associations between the criterion variables and parent/carer-reported experience of SDM controlling for demographics and using conventional (i.e., standard/simple single-level) logistic regression analysis (model one). This unadjusted model included only individual/family level variables and did not consider the service level influence. Due to the nested nature of the dataset, a null model was fitted using the CAMHS Service ID and revealed an intraclass correlation coefficient (ICC) of almost 48% (ICC = 0.479) of the variance of SDM being explained at the service-level. As a result, families attending the same CAMHS site may share similar experiences biassing estimates of standard errors when examining the effect of services. Consequently, we investigated the associations between the criterion variables and parent/carer-reported experience of SDM using a multilevel mixed-effect logistic regression analysis (model two). The results of associations are shown as odds ratios (ORs) with a 95% confidence intervals (CIs). A two-sided *p*-value of <0.05 was considered significant (45).

To address the aims of the study, model one was compared to model two. Researchers argue that estimates of specific effects (e.g., OR) provide insufficient information if they are not accompanied by measures of general contextual effects (i.e., area under the receiver operating characteristic curve, AUC) (46). In line with Merlo et al. (46) recommendations for multilevel logistic regression of discriminatory accuracy, the AUC was estimated and compared. Therefore, the higher the AUC, the better the model was at distinguishing between lower and higher quality experiences of SDM (47). Additionally, the Akaike information criteria (AIC) was used as a measure of goodness of fit of the models (48). STATA (v 11) was used to conduct the analyses (49).

#### Ethical Considerations

The primary author obtained the necessary permission to conduct secondary analysis on routinely collected administrative data from CAMHS. Data was received in an anonymous format and only accessible via a password-protected server. As a result, this study did not require any formal institutional ethical approvals (50, 51), and we received permission to proceed with our analysis from the University research ethics committee.

## Results

The sample included in the analysis was composed of *N* = 3,175 cases of CYP accessing care from 58 CAMHS offered by the National Health Services in England (see Table 1).

### Frequency of Parent/Carer Experience of SDM at CAMHS

Overall, 69.23% (2,198/3,175) of the parents/carers reported experiencing higher levels of SDM. For each of the four items on the SDM measure, over 90% of parents/carers reported that it was “true” the healthcare provider related to them in ways consistent with SDM.

### Model 1: Factors Associated With Parent/Carer Experience of SDM (Unadjusted)

Model one was statistically significant, χ^2^ (32) = 220.48, *p* <0.05, suggesting associations between ethnicity, relationship to the child, presence of conduct problems or learning difficulties and parent/carer experience of SDM were observed. The regression model explained almost 6% of the individual level variance in SDM (*R*^2^ = 0.056). More specifically, Asian parents/carers (OR = 1.95, 95% CI [1.4, 2.73]) and parents/carers having children with learning difficulties (OR = 1.45, 95% CI [1.06, 1.97]) were more likely to report higher levels of SDM. However, having both parents/carers involved in the child's care and treatment decisions (OR = 0.3, 95% CI [0.21, 0.44]) and being a parent/carer of a child or young person experiencing conduct problems (OR = 0.78, 95% CI [0.63, 0.98]) were associated with lower levels of SDM. No other significant associations were identified. Results of the model are presented in Table 2.

**Table 2:** Regression coefficients, variation and fit indices across fitted models.

	**Simple logistic regression analysis (unadjusted)**		**Multilevel logistic regression analysis (adjusted)**	
**Parameters**	**Model 1^**a**^**		**Model 2^**b**^**	
	**OR (SE)**	**95% CI**	**OR (SE)**	**95% CI**
**Demographics**				
Age of child	1.02 (0.09)	0.86–1.21	0.96 (0.09)	0.79–1.16
Gender of child				
male vs. female	1.03 (0.88)	0.87–1.22	1.06 (0.1)	0.85–1.27
Ethnicity of child				
Mix vs. white	0.94 (0.16)	0.67–1.32	1.02 (2)	0.7–1.49
Asian vs. white	1.95 (0.33)^**^	1.4–2.73	1.43 (0.28)	0.97–2.11
Black vs. white	1 (0.19)	0.69–1.46	0.81 (0.16)	0.55–1.2
Other vs. white	1.19 (0.14)	0.94–1.51	1.07 (0.15)	0.81–1.41
Relationship to child				
Father vs. mother	1.1 (0.19)	0.78–1.55	1.05 (0.19)	0.73–1.5
Both parents vs. mother	0.3 (0.56)^**^	0.21–0.44	0.75 (0.18)	0.47–1.2
Other vs. mother	1 (0.292)	0.57–1.77	1.16 (0.35)	0.64–2.1
**Psychosocial difficulties**				
Separation anxiety	1.12 (0.11)	0.91–1.36	1.21 (0.14)	0.97–1.51
Social anxiety	0.99 (0.1)	0.81–1.21	0.97 (0.11)	0.78–1.21
General anxiety	0.85 (0.08)	0.7–1.02	0.84 (0.09)	0.69–1.03
OCD	0.94 (0.12)	0.74–1.2	0.1 (0.14)	0.77–1.3
Panic disorder	1.06 (0.12)	0.85–1.33	0.1 (0.12)	0.78–1.3
Agoraphobia	0.98 (0.13)	0.76–1.28	1.02 (0.15)	0.77–1.35
Depression	0.96 (0.09)	0.79–1.16	0.96 (0.1)	0.78–1.17
Self-harm	0.94 (0.11)	0.75–1.19	0.87 (0.11)	0.68–1.12
ADHD	0.88 (0.1)	0.7–1.11	0.91 (0.12)	0.71–1.16
Conduct disorders	0.78 (0.09)^**^	0.63–0.98	0.75 (0.09)^**^	0.59–0.94
Difficult to manage	1.09 (0.12)	0.88–1.34	1.14 (0.14)	0.9–1.44
Family problems	0.99 (0.11)	0.8–1.23	0.98 (0.12)	0.78–1.24
Attachment problems	1.07 (0.12)	0.85–1.34	1.16 (0.15)	0.91–1.5
Peer problems	0.89 (0.09)	0.73–1.07	0.88 (0.09)	0.72–1.9
Other	0.87 (0.09)	0.72–1.06	0.82 (0.09)	0.66–1.02
**Additional problems**				
Learning difficulties	1.45 (0.23)^**^	1.06–1.98	1.16 (0.19)	0.84–1.6
Autism	0.91 (0.12)	0.7–1.18	0.9 (0.13)	0.68–1.19
Child in need	0.74 (0.12)	0.84–1.03	0.71 (0.13)	0.5–1
Experience of abuse	0.87 (0.12)	0.67–1.14	0.88 (0.13)	0.66–1.18
Parental health issues	1.04 (0.11)	0.85–1.27	1.12 (0.13)	0.89–1.4
Financial difficulties	1.12 (0.18)	0.81–1.52	1.01 (0.17)	0.72–1.42
Other	1.18 (0.13)	0.95–1.47	1.2 (0.14)	0.95–1.52
**Impact**				
Home	1.09 (0.1)	0.9–1.31	1.04 (0.11)	0.85–1.28
School/work	0.9 (0.09)	0.75–1.09	0.84 (0.09)	0.68–1.03
Community	1.01 (0.12)	0.8–1.26	1.09 (0.14)	0.85–1.4
Service engagement	1.14 (0.17)	0.85–1.54	1.05 (0.17)	0.76–1.44
Amount of variance				
Pseudo R_sq (%)	0.06 (6)	
ICC (%)			0.45 (45)	
AUC	0.6511		0.7391	
AUC change^*^			0.0088	
Goodness of fit				
AIC	3756.85		3433.82	
AIC change^*^			322.18	

*AIC, Akaike information criteria; AUC, Area under the receiving curve; ICC, Intraclass correlation coefficient; OR, Odds ratio; CI, Confidence intervals; OCD, Obsessive compulsive disorder; ADHD, Attention deficit hyperactivity disorder*.

*N = 3,175*.

** *p > 0.05*.

* *change in relation to the previous model*.

a *Model 1: SDM + demographics, MH difficulties, additional problems and impact (unadjusted).*

b *Model 2: SDM + demographics, MH difficulties additional complex problems and impact (adjusted)*.

### Model 2: Factors Associated With Parent/Carer Experience of SDM (Adjusted)

When adjusting for service level factors, χ^2^ (35) = 45.60, *p* <0.05, only the presence of conduct problems was found to be statistically significant and predicted lower levels of SDM (OR = 0.29, 95% CI = [0.52, 0.58]). No other significant associations were identified.

### Model Diagnostics

It was observed that the adjusted model (model two) accounted for higher discriminatory accuracy in parents/carers experience of SDM than the unadjusted model (AUC change of 0.0088). This indicated that the added value of potential service level data introduced a higher chance of that model being able to distinguish between parent/carer experience of higher or lower levels of SDM. Model two also had the lowest AIC and as such was selected as the model that best fitted the current dataset. AUC and AIC scores are reported in Table 2.

## Discussion

The current study first aimed to statistically describe parents/carers experience of SDM at CAMHS. In addition we examined associations between parent/carer reported experience of SDM and clinician's perceptions of CYP psychosocial difficulties, additional complex problems and the impact of the psychosocial difficulties.

The results of this study indicated that almost 70% of parents/carers reported experiencing higher levels of SDM (4 out of 4) at CAMHS which aligns with the high proportion of self-report SDM in the previous literature (12–18). Although parents/carers in the current study reported high levels of SDM, it may not be sufficient to represent the complex nature of SDM in a triad (9), since researchers generally report several barriers to successful SDM in CAMHS (34, 52). Therefore, this raises further questions of whether we are accurately capturing SDM with existing self-report measures in CAMHS. One possible explanation may be that not all service users want to be involved in healthcare decision-making (53). However, it must be noted that studies usually represent specific decisions, for example, parents facing challenges during medicinal decision-making (29). Also, with the increasing promotion for CYP to be actively involved in their care and treatment decisions (54), future studies can further explore how decision type and number of decision-makers affect parent/carer levels of SDM in CAMHS. Nonetheless, the current findings add to the existing knowledge base by reporting frequency of parental SDM in CAMHS in England, and represents a sample experiencing a wider range of clinical characteristics and age range beyond those commonly reported in the previous studies. The current findings also advance the observed SDM trend reported in the UK (19), by providing the most recent statistics in a specific CYP population.

To address the second aim of this study, only individual-level data was used in model one. We identified significant associations between ethnicity, relationship to the child, presence of conduct problems and learning difficulties and SDM. This aligns with previous research which demonstrated that higher levels of psychosocial difficulties were associated with lower experiences of SDM among parents (26). More specifically, the more severe the behavioral difficulties the lower the level of parent/carer SDM was reported (15). However, due to the cross-sectional nature of the study it was not feasible to determine the direction of the relationship. Although previous studies found associations between other psychosocial difficulties (e.g., anxiety) and level of impact and parents/carers SDM (13, 26), these findings were not replicated in the current sample. One possible explanation for this might be that previous samples used continuous variables for the clinical characteristics and therefore captured severity, whereas the current study explored the mere presence of the problem as measured on a dichotomous scale which limits the capacity to explain variability (55). Nonetheless the current study builds on previous research by highlighting the importance of taking into account the additional complex factors such as learning difficulties. The positive relationship could be as a result of the existing policy guidelines for SDM among people with learning difficulties which recommend the involvement of family members to support the patient (56).

For the third aim of the study, model two was selected as the model that best fitted the current dataset and included a combination of clinical and demographic characteristics. This is consistent with the general SDM literature indicating the influence of both clinical and demographic characteristics on SDM among service users. For example, systematic reviews have consistently reported demographic and health status as influencing factors (33, 35). Further investigations confirmed that when accounting for service-level data the model had a better chance of distinguishing between parents/carers experience of SDM. This also aligns with the existing literature confirming the importance of higher-level factors such as time constraints at the clinics, motivation and skills of the clinician, and available resources (21, 33–35, 52). For the most part, these findings suggest that targeting factors at individual and larger ecological levels will remain important. However, failing to acknowledge the service user characteristics and efficacy downplays the important role that individuals may play in contributing to their own care and treatment. At the same time, relying too heavily on only individual-level change neglects the role that environments and context have in influencing individuals' decisions and behaviors.

Although model one revealed that the involvement of both parents/carers in the CYP's care and treatment resulted in lower levels of SDM, the area of triad relationships in SDM in CAMHS is yet to shed light on this phenomenon. However, this finding is not surprising as researchers in adult healthcare suggest that the involvement of an additional family member increases the complexity of the interactional dynamics (1). Similarly, parents identifying as Asian in the current sample were associated with higher levels of experiencing SDM. This is surprising because research shows that minority ethnic groups (e.g., Blacks and Hispanics) report lower experiences of SDM than White Caucasians families (27). Therefore, further investigations using qualitative designs and purposive samples are needed.

### Future Directions

The findings of this study suggest that policies and interventions to improve SDM in CAMHS should target both services and individuals. However, to give further insight into identifying target groups (e.g., parents/carers of CYP with conduct problems), more information is needed. Therefore, as recommended by other researchers, future research including specific service level variables, such as population size of the service or number of clinicians will further enhance our understanding. Additionally, it may be just as important to identify clinician-level variables such as years of experience or area of expertise that may further explain variation in experiences of SDM. Hence, a three-level analysis will help to inform our knowledge of this phenomenon. As confirmed by this study, more qualitative research is needed to help inform the SDM predictor variables (for example, presence of problem vs. severity of the problem vs. impact) in order to capture critical thresholds that may influence parent/carer experience of SDM. Another recommendation for future research would be to repeat this study using a longitudinal sample to capture the directional nature of the variables and infer causality. Lastly, similar to Edbrooke-Childs et al. (26), it is recommended that future studies include child- and clinician- reported SDM to fully capture the triad relationship. These are important factors that can possibly influence parent/carer level of involvement (9).

### Strengths and Limitations

First, this study incorporates a variety of observer-reported predictor variables beyond psychosocial difficulties while the majority of previous studies focused mainly on the self-report severity of the CYP mental health. Additionally, using a broad range of psychosocial difficulties added to the potential to target specific disorders such as types of anxieties and mood problems that could influence SDM, as opposed to categorizing difficulties into broader groups of anxiety and depression. Second, considering the nested nature of the data and utilizing an innovative multilevel analytic approach highlighted the important potential influence of service level factors on an individual level experience of SDM. This is crucial to the study of SDM as without this knowledge, interventions and policies may be developed and implemented without taking this contextual level variation into account. This can result in the inefficient allocation of government funds and unproductive use of both the clinician's and service user's time.

In spite of these strengths, the findings of this study should be considered as exploratory and interpreted with caution due to several design and measurement limitations. The current data represents only a cross-section of the population. The items used to calculate the composite SDM score were taken from the self-report ESQ and therefore may be prone to bias. Although this measure has been used in previous studies as a measure of SDM (26), a high percentage of the sample scored 4 out of 4 suggesting ceiling effects which are common in these types of measures (57). Considering this as an exploratory study, by dichotomizing the composite measure we were better able to address the aim of our study to identify the frequency of “higher quality” SDM experiences. In addition, dichotomising the measure was based on the decision to be consistent with previous research (15), and therefore aid with comparisons. The decision was also based on the limitations of previous studies reporting challenges with the low to high spectrum and its inability to determine parents/carers' “full” experience of SDM (14).

Another limitation is the low representativeness of fathers and ethnic minorities in the sample due to the constraints of conducting secondary analysis of routinely collected data. This in itself is a limitation as the data was not collected under controlled conditions and there may be variations among sites on instruments used and how data was collected. Another limitation of the dataset, with implications for the analysis and interpretation, was the pooled categorisation of clinical characteristics (e.g., selective mutism and Gender Identity Disorder) which represented <10% of the sample. Together these low frequency problems accounted for over 50% of the total sample. This may influence the study's findings raising assumptions that these characteristics influence parent/carer experience of SDM in the same way. Despite the study's limitations it remains one of the few quantitative studies to examine parent/carer SDM in CAMHS in England and the knowledge gained can be used as a basis for future research.

## Conclusion

In summary, this study has highlighted the need for using a multilevel approach to promoting and implementing SDM interventions in CAMHS, as suggested by the high service level variation (ICC = 0.48) in parent/carer-reported SDM. This identifies CAMHS sites to be a potential target for effective intervention. However, the findings of this study suggest that more research is needed if data is to be modeled in this way. Ethnicity, learning difficulties, relationship to the child and conduct disorders were the only potential service user level factors that were associated with SDM in the simple logistic regression and the presence of conduct disorders remained the only significant predictor variable when accounting for service level factors. Future analyses of SDM could aim to utilize more detailed measures of SDM and include clinician level factors, such as the clinician's years of experience, and service level factors, such as population size, to help explain the variability in SDM. Future research could also include clinician and young people experience of SDM to further understand the triad relationship. Nonetheless, this exploratory study highlights the evident influence of service-level factors on parent/carer experience of SDM and suggests that families with children experiencing conduct problems could be targeted for additional support if they are to be involved in the SDM process.

## Data Availability Statement

The data analysed in this study is subject to the following licences/restrictions: Restrictions apply to the availability of these data, which were used under licence for this study. Data are available https://www.corc.uk.net/media/1883/request-for-use-of-corc-dataset-27-09-2018.doc with the permission of the Child Outcomes Research Consortium. Requests to access these datasets should be directed to https://www.corc.uk.net/media/1883/request-for-use-of-corc-dataset-27-09-2018.doc with the permission of the Child Outcomes Research Consortium.

## Ethics Statement

Ethical review and approval was not required for the study on human participants in accordance with the local legislation and institutional requirements. Written informed consent from the participants' legal guardian/next of kin was not required to participate in this study in accordance with the national legislation and the institutional requirements.

## Author Contributions

All authors contributed to the conception and design of the study. Analysis and interpretation of the data were conducted by the primary author and supported by the other authors. The primary author drafted the manuscript and the other authors contributed to the editing and refinement of the article before submission. All authors contributed to the article and approved the submitted version.

## Conflict of Interest

The authors declare that the research was conducted in the absence of any commercial or financial relationships that could be construed as a potential conflict of interest.

## Abbreviations

CAMHS: Child and Adolescent Mental Health Services
CVT: Current View Tool
CYP: Children and young people
ESQ: Experience of service questionnaire
SDM: Shared decision making.
